# Cross-border outbreak of extensively drug-resistant tuberculosis linked to a university in Romania

**DOI:** 10.1017/S095026881800047X

**Published:** 2018-05-17

**Authors:** O. Popovici, Ph. Monk, D. Chemtob, D. Chiotan, P.J. Freidlin, R. Groenheit, M. Haanperä, D. Homorodean, M. Mansjö, E. Robinson, E. Rorman, G. Smith, H. Soini, M. J. Van Der Werf

**Affiliations:** 1 National Institute of Public Health, National Centre for Surveillance and Control of Communicable Diseases, Bucuresti, Romania; 2 Public Health England East Midlands Centre, Nottingham, England; 3 Ministry of Health, Department of Tuberculosis (TB) and AIDS and National TB Programme manager, Jerusalem, Israel; 4 Hebrew University-Hadassah Faculty of Medicine, School of Public Health and Community Medicine, Jerusalem, Israel; 5 National Institute of Pulmonology “Marius Nasta”, Bucuresti, Romania; 6 Ministry of Health, Public Health Services, National Public Health Laboratories, Tel Aviv, Israel; 7 National Center for Mycobacteria, Tel Aviv, Israel; 8 Public Health Agency of Sweden, WHO Supranational TB Reference Laboratory, Solna, Sweden; 9 Department of Health Security, National Institute for Health and Welfare, Helsinki, Finland; 10 National Reference Laboratory for Tuberculosis Cluj-Napoca, Romania; 11 Public Health England, National Infection Service, Public Health Laboratory, Birmingham, England; 12 Ministry of Health, Public Health Services, Director National Public Health Laboratories, Tel Aviv, Israel; 13 Public Health England, Director National Mycobacteriology Reference Service, National Infection Service, Public Health Laboratory, Birmingham, England; 14 European Centre for Disease Prevention and Control, Stockholm, Sweden

**Keywords:** Contact tracing, extensively drug-resistant, tuberculosis, whole genome sequencing

## Abstract

Extensively drug-resistant (XDR) tuberculosis (TB) poses a threat to public health due to its complicated, expensive and often unsuccessful treatment. A cluster of three XDR TB cases was detected among foreign medical students of a Romanian university. The contact investigations included tuberculin skin testing or interferon gamma release assay, chest X-ray, sputum smear microscopy, culture, drug susceptibility testing, genotyping and whole-genome sequencing (WGS), and were addressed to students, personnel of the university, family members or other close contacts of the cases. These investigations increased the total number of cases to seven. All confirmed cases shared a very similar WGS profile. Two more cases were epidemiologically linked, but no laboratory confirmation exists. Despite all the efforts done, the source of the outbreak was not identified, but the transmission was controlled. The investigation was conducted by a team including epidemiologists and microbiologists from five countries (Finland, Israel, Romania, Sweden and the UK) and from the European Centre for Disease Prevention and Control. Our report shows how countries can collaborate to control the spread of XDR TB by exchanging information about cases and their contacts to enable identification of additional cases and transmission and to perform the source investigation.

## Introduction

Extensively drug-resistant (XDR) tuberculosis (TB) was first described in a study using samples from different geographical areas from the period 2000–2004 [1]. According to the World Health Organization (WHO) case definition, XDR TB strains are resistant to most anti TB drugs, more specifically to isoniazid and rifampicin, to at least one fluoroquinolone and to at least one of the injectable drugs amikacin, capreomycin or kanamycin [2]. Treatment of patients with XDR TB is difficult and of all XDR TB cases diagnosed in 2012 in the European Union (EU) only 20% were successfully treated [3].

Following established procedures [4], a cross-border cluster of XDR TB, including one Israeli and two British medical students, was notified by the Romanian National Centre for Communicable Diseases Surveillance and Control (NCCDSC) to the European Centre for Disease Prevention and Control (ECDC) on October 10, 2016. Thereafter, an international cluster investigation was initiated, including epidemiological investigation and genomic typing by whole genome sequencing (WGS). Contact investigations were implemented in all countries involved, i.e. Finland, Israel, Romania and the UK. Here we report on the results of contact investigations and the efforts to identify the source of this outbreak.

## Methods

The initial cluster was defined based on the drug susceptibility pattern (XDR TB) and place criteria (same university). The WGS results were used to precisely delimit the outbreak.

The outbreak investigation included case notifications, contact investigations and source investigation.

### Case notifications

Cases were notified to countries through the Early Warning and Response System (EWRS), WHO–International Health Regulation (IHR) or directly, to the ECDC TB network.

### Contact investigations

Each country with a case of XDR TB linked to the cluster performed a contact investigation following national procedures. Tuberculin skin testing (TST) (five units (U) purified protein derivative (PPD)) or interferon gamma release assays (IGRA) were used to identify TB infection. In Israel, a positive TST is defined as five mm or more induration (palpable, raised, hardened area or swelling) for close contacts of an active TB case and 10 mm or more for healthcare worker screening if there was no close contact with a TB case. According to the Romanian guidelines, a positive TST result is defined as an induration of at least 10 mm in immune-competent and HIV negative persons and at least five mm in HIV positives and other immune-suppressed. In the UK, the IGRA (QuantiFERON^®^-TB Gold In-Tube Test) was used. For the diagnosis of TB disease Xpert MTB/RIF (Cepheid, Sunnyvale, CA, USA), chest X-ray, sputum smear microscopy and culture (Löwenstein-Jensen) were applied. Xpert MTB/RIF and phenotypic methods were used for the detection of drug resistance. In addition, spoligotyping, 24 loci Mycobacterial Interspersed Repetitive Units Variable Number Tandem Repeat (MIRU-VNTR) and whole-genome sequencing (WGS) were used to study strain relatedness and transmission. WGS was performed in the country of residence of the TB cases, Finland, Israel and the UK, and the conclusive analysis was performed by the WHO Supranational Reference Laboratory (SRL) for TB in Stockholm. The WGS methodology used is summarised in Table 1.

**Table 1.** Whole-genome sequencing methodology used for sequencing of the strains in the extensive drug-resistant tuberculosis outbreak investigation

Sequencing service, Organisation and country	Sequencing platform; manufacturer and model	Software for computational pipeline – peer-reviewed publication	Criteria for quality assessment/quality control of assembly	Genome assembly. Reference mapping or *de novo*	Variant calling software	Relatedness	Validation	Quality assurance/control	In routine clinical use?	In clinical use for selected isolates	In use for research	Isolates sequenced/analysed for this investigation
National Institute for Health and Welfare, Finland	MiSeq	Mostly. RIDOM SeqSphere	Reference coverage (depth and %) mapped reads	Mapping and Burrows-Wheeler Aligner de novo (Velvet)	RIDOM Seqsphere	Implementation of methodology ongoing			No	MDR strains and isolates included in contact investigations	Yes	Case 6
National Public Health Laboratory Tel Aviv, Israel	Illumina Hiseq 4000	BioNumerics 7.6, Applied Maths	Reference coverage (depth and %) mapped reads	Mapping and de novo	BioNumerics 7.6	Implementation of cgMLST in BioNumerics, still under evaluation and validation		In-house controls (Accred Qual Assur (2011)16 : 623–635)	No	Yes	Yes	Cases 1 and 5
Public Health Agency of Sweden, Sweden	Ion Torrent	No	Reference coverage (depth and %), amount of ambiguous calls	de novo (CLC Assembly Cell v 4.4.2)	CLC Assembly Cell v 4.4.2	In house-script	Swedac (Sweden's national accreditation body). In progress	ERLTB-Net	Yes	Not applicable	Yes	Isolates from Romanian patients
Public Health England/Modernising Medical Microbiology Consortium, University of Oxford, UK	Illumina MiSeq	No	Reference coverage %, mapped reads	Reference mapping (Stampy-open source)	Custom	Elephant walk (custom)	United Kingdom Accreditation Service (UKAS) inspection against ISO15189 (2012) in progress	IQC and IQA Global Microbial Identifier proficiency testing; UK NEQAS for mycobacterial diagnostics	Yes	Not applicable	Yes	Cases 2, 3, 4 and 7

IQC, internal quality control; IQA, internal quality assurance.

### Source investigation

To identify the ‘source’ of the outbreak (the case that started the outbreak), all pre-XDR and XDR TB strains from patients diagnosed up to 2016 in the county where the university is located, were identified and sub-cultured in the National Reference Laboratory (NRL) for Tuberculosis in Cluj-Napoca. Those which could be sub-cultured were subjected to WGS analysis at the Swedish NRL (WHO SRL for TB for Romania) and compared with the outbreak strain.

The WGS results of the outbreak strain were compared with WGS patterns in different databases available to the outbreak investigation team. The SRL for TB in Stockholm compared the WGS results of the strains belonging to the cluster with all isolates in the national Swedish WGS database (more than 1300 isolates). In Sweden, the majority of TB patients originate from Africa and in particular Somalia (~24% of all TB-patients). The UK compared the WGS patterns of the outbreak strains with the information in their WGS database containing approximately 14 000 *Mycobacterium tuberculosis* complex sequences obtained from over 15 countries and all continents. Israel compared the WGS patterns with their new WGS database which includes a limited number of strain data and with another database of all new isolates diagnosed in Israel since 2008. This database contains some 2700 genotypic patterns characterised by spoligotyping and 24 loci MIRU-VNTR. Finland compared the spoligotyping and MIRU-VNTR patterns of the outbreak strain with the strains in the Finnish spoligotyping and MIRU-VNTR database which includes more than 4000 strains isolated in Finland. Since some of the cases in the cluster have a link with Somalia, the outbreak strain was also compared with the patterns of strains belonging to an outbreak of multidrug-resistant (MDR) TB among migrants from the Horn of Africa [5, 6]. Finally, the DNA fingerprint of the outbreak strain was compared to large databases based on MIRU-VNTR data (>11 000 datasets) and WGS data (>8500 datasets) held by the Research Center Borstel in Germany that comprise multiple strain collections from different world regions.

Since the source could be a patient of Romanian origin treated in Hungary, as the county where the university is located is neighbouring Hungary, ECDC checked The European Surveillance System (TESSy) database for MDR or XDR TB cases with Romanian origin being treated in Hungary between 2010 and 2015 to identify a possible source case.

### Epidemiological contact investigation

All identified XDR TB cases belonging to the cluster were interviewed to identify epidemiological links. Each country used its own procedures and interview formats for the epidemiological contact investigation.

## Results

The outbreak included a total number of seven confirmed XDR TB cases. The first case in the cluster was an Israeli medical student who had returned home after the first year of studies (started on 1 October 2014) at a university in Romania and was diagnosed with extra-pulmonary XDR TB in Israel in October 2015 (case 1). She had a documented negative TST in July 2014, before arriving at the university in Romania. After returning to Israel, the TST had converted to positive (13 mm) and pleural TB disease was diagnosed. The MIRU-VNTR pattern of the TB strain causing the disease had not been identified in Israel before. The case was notified to the Romanian NCCDSC on 20 October 2015, by the IHR National Focal Point (NFP) for Israel.

### Contact investigations coordinated by Romania

As a result of the case notification, the Romanian authorities initiated contact tracing in October 2015 and identified 59 close contacts among students, teachers and other personnel. A person was considered a close contact if being at conversation distance with case 1 for a cumulative period of at least 8 h in a closed space, starting from February 2015 (the presumed month of onset for case 1).

The identified contacts were of 14 nationalities: Romania (17), Finland (12), UK (nine), Germany (four), Israel (three), Nigeria (three), Sweden (three), Republic of Mauritius (two), Austria (one), Hungary (one), Italy (one), Palestinian Authority (one), Poland (one) and the United Arab Emirates (one). All but two contacts showed normal chest X-ray results during the screening. One contact (case 2) was diagnosed with TB in Romania in October 2015 by chest X-ray and sputum smear microscopy, and by culture (Löwenstein–Jensen) in January 2016, and thereafter returned to the UK for further investigations and initiation of XDR TB treatment. In Romania, the laboratory result which confirmed the XDR TB was available in March 2016. In addition, one of the contacts, a Finnish student, was diagnosed with drug-sensitive TB in his country of origin (unrelated event).

According to the Romanian guidelines, TST was performed in contacts who were aged under 19 years. Of the two investigated contacts, one had a negative (four mm) and one a positive (20 mm) TST result (case 2).

At the end of September 2016, an additional case of XDR TB related to this cluster (case 3) was notified by the UK to the National Institute of Pulmonology (NIP) in Bucharest, Romania, which further reported to the Romanian NCCDSC. The case had no symptoms and was picked up in the UK through contact tracing, as a contact of case 2. He was also one of the 59 contacts of case 1 traced in October 2015 in Romania, at which time he had a negative chest X-ray. The UK further informed Romania, in October 2016 that this case with pulmonary TB had a positive culture after 6 weeks. The infectious risk was considered very low because of the negative microscopy result and because it took such a long time to grow the culture.

Following the identification of case 3, the Romanian NCCDSC informed ECDC about a cluster of three XDR TB cases linked to a university. On 12 October 2016, Romanian authorities posted the first message related to a public health alert, using the international channels, followed by bilateral messages and by updates to countries involved. On 19 October 2016, the Israeli authorities shared the Drug Susceptibility Testing (DST) results of case 1 with the Romanian NCCDSC and ECDC. A week later, on 26 October 2016, the Romanian NCCDSC shared the MIRU-VNTR 24 loci typing data of the XDR TB strains isolated in Israel (case 1) and UK (cases 2 and 3). The two UK strains had the same MIRU-VNTR pattern, whereas the MIRU-VNTR of the Israeli strain showed one repeat difference in locus 580. This small difference indicates a close relation between the strains. Since Israel used another method for interpreting the results of locus 580 than the UK that may produce results that differ in one repeat, it is also possible that the strains were identical [7].

The notification of case 3 triggered the resumption of the epidemiological investigations in Romania and all the contacts were re-evaluated. The contact tracing initiated on 4 October 2016 increased the total number of contacts in Romania to 97: 65 students of which 11 from Romania and 32 university employees. From the 38 newly identified contacts, 13 were students and 25 were university employees. Ten contacts identified in the first contact investigation had already left Romania; therefore the results of their first screening were communicated to the authorities in their countries of origin. Out of 97 contacts, 92 were screened by chest X-ray (89 in Romania, three in other countries), one refused the investigation and four were not eligible: cases 2, 3, one German student detected with Hodgkin's lymphoma at chest X-ray done in Romania in October 2015, and the already mentioned Finnish case, with a drug-susceptible strain. Two contacts were identified as suspected TB. Sputum examinations were initiated for 63 contacts and all were negative for acid-fast bacilli on sputum microscopy, Xpert MTB/RIF and culture. For 34 contacts no laboratory investigations were undertaken; these include cases 2, 3, the above-mentioned Finnish case and 31 university employees. For all eligible 94 contacts TST was performed (92 in Romania, one in France and one in Italy) based on a new protocol for tracing and follow-up of contacts of an XDR TB case created by the NIP in October 2016. The TST investigation showed 51 negative and 43 positive (⩾10 mm) results. For three contacts, TST was not performed: cases 2, 3 and the same Finnish case. In the initial contact investigation in October 2015, university personnel were not investigated by TST since they were all above 19 years of age. However, university personnel were included in the TST investigation in October 2016.

For the two contacts with suspected lesions on chest X-ray, computer-tomography (CT) examination was performed and one contact with Israeli citizenship (case 5) was diagnosed with TB-specific lesions in October 2016. The student decided to return to Israel for further investigations and treatment.

The results of contact investigations were sent via the EWRS and the WHO–IHR to all the countries of origin.

The TST was repeated in January 2017 for 50 contacts with negative results in October 2016: one in Germany, one in France (university personnel) and 48 in Romania. Six contacts were positive, so a chest X-ray was done, all with a negative result. A single contact was not investigated (university personnel) because the person was undergoing cancer treatment.

Contacts will be followed until October 2018.

### Contact investigations in the UK

The contact investigation around case 2 included screening by IGRA of the eight family members. The initial IGRA test was positive for two family members. One of these was diagnosed with XDR TB in March 2016 (case 4). Another family member tested IGRA positive but had a normal chest X-ray and no TB. Of the six family members with an initial negative IGRA result, five converted to positive. Two (cases 7 and 8) of those were diagnosed with TB, the others remained chest X-ray negative. Case 8 was not bacteriologically confirmed. Another family member had six consistently negative IGRA tests between March 2016 and August 2017.

A further contact of case 4 was diagnosed with pleural TB (case 9) on 10 February 2017 and started treatment on 8 March 2017. He had no confirmatory bacteriology. Public Health England (PHE) notified the Romanian authorities about case 4.

### Contact investigations in Finland

On 25 October 2016, Finland posted an EWRS message on an XDR TB case (case 6) notified in August 2016: male, Somalian origin, but born in Finland. A sputum specimen taken on 12 August 2016 was smear microscopy and PCR positive, and later *M. tuberculosis* was isolated. The patient had cavitary TB disease. Case 6 was a close contact of cases 4 and 2 and shared considerable time with smear-positive case 4.

The contact tracing in Finland identified 12 contacts: four children and eight adults (parents and friends). IGRA tests and chest X-ray examinations were performed for the children and all were negative. Adult contacts were examined only by chest X-ray and were all negative.

### Contact investigations in Israel

Shortly after the notification of case 1 (23 August 2015) was made to the District Health Office, an epidemiological enquiry took place. TST was performed on the family members (parents, four sisters and one brother). TST of the two parents was 15 mm, but their IGRA test was negative. TST was zero mm for all siblings, except for one with 10 mm induration, but with a negative IGRA test. Since the IGRA tests were negative, no further investigations were performed.

Contact investigations for case 5 were performed following the notification on 30 October 2016. Twenty-four close contacts were identified and TST was performed for all of them. Nine persons were found TST positive; their chest X-rays were negative.

### Genotyping and WGS investigation

**Fig. 1.**
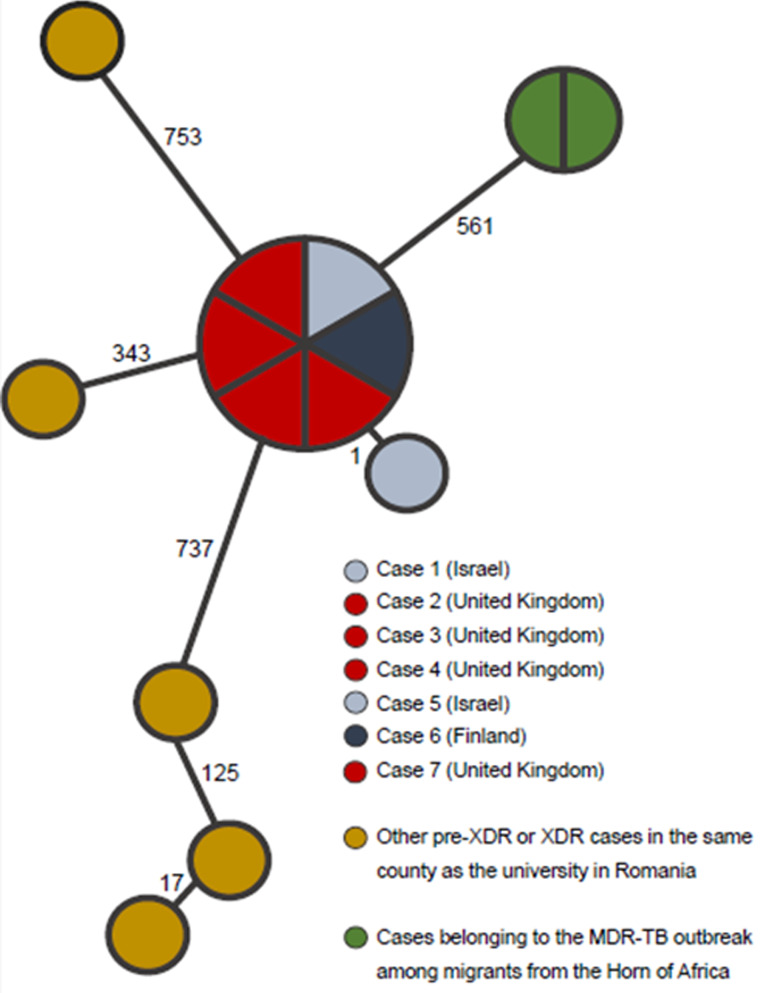
Illustration of a Minimum Spanning Tree with SNP differences indicated on the branches (core genome included in the analysis = 92.93%). Source: The WHO Supranational Reference Laboratory (SRL) for TB in Stockholm, Sweden.

WGS investigations showed that the isolates from cases 2, 3, 4, 5, 6 and 7 had identical WGS patterns, with zero single nucleotide polymorphisms (SNPs) (Fig. 1). The isolate from the index case (case 1) showed 1 SNP difference. The isolates shared the same novel mutation, Ile588Val, in the *rpoB* gene.

### Source investigation

To assess whether a strain circulating within the county where the university is located was the source of the outbreak, all pre-XDR and XDR TB strains from patients diagnosed up to 2016 in the county were identified. Five of six strains from 2015 and 2016 showed growth and were subjected to WGS analysis, none of the strains from patients diagnosed before 2015 showed growth. None of the strains subjected to WGS showed relatedness to the outbreak strain. The closest related strain differed by 343 SNPs from the outbreak strain.

Comparison of the outbreak strain with the isolates in the Swedish WGS database showed no close relatedness. All strains in the database differed more than 25 SNPs from the outbreak strain. Comparison of the outbreak strain with the UK WGS database showed that the nearest strain was 140 SNPs away. In the Israeli spoligotyping and 24 loci, MIRU-VNTR databases no matches were found with the outbreak strain. In the Finnish spoligotyping and MIRU-VNTR database one strain had a very similar MIRU-VNTR type compared to the XDR TB outbreak strain. This strain was isolated from a Romanian patient who studied in Finland in 2010 but was susceptible to all tested drugs (isoniazid, rifampicin, pyrazinamide, ethambutol and streptomycin). WGS data of this strain were not available. The patient was diagnosed with pulmonary TB in Finland in April 2010 and started treatment which was continued in Romania in June 2010, finished in October 2010 and evaluated as cured in June 2011. The patient studied in Romania at a different university than cases 1 and 2, and there was no apparent link with the cluster.

The comparison of the outbreak strain with the strains from an outbreak of MDR TB among migrants from the Horn of Africa showed that the strains from the MDR TB outbreak were not related to the XDR TB outbreak (561 SNPs distance) (Fig. 1).

The large databases held by the Research Center Borstel in Germany did not contain strains with an identical MIRU-VNTR pattern (24-loci) or Core Genome Multilocus Sequence Typing (cg-MLST) scheme with less than 12 allele differences with the outbreak strain.

ECDC did not identify any MDR or XDR TB case with Romanian origin being treated in Hungary between 2010 and 2015 in the TESSy database.

### Epidemiological contact investigation

**Fig. 2.**
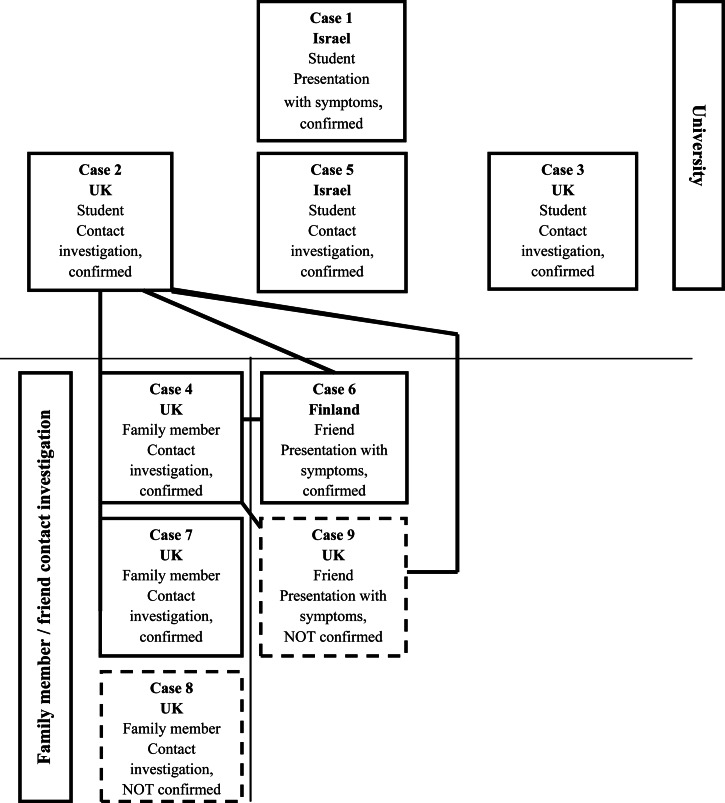
Epidemiological links between the seven confirmed extensively drug-resistant tuberculosis cases in the outbreak and two other epi-linked cases in UK contacts.

Epidemiological investigation revealed that cases 1–3 were in contact with each other at the university. Cases 2 and 3 shared accommodation in Romania. Case 4 was a family member of case 2 and shared a room in their house in the UK. Case 6 was a close friend of case 4 and shared considerable time and had also contact with case 2. Case 6 had never visited Romania. Case 7 was a family member of case 2 (Fig. 2).

More details about the XDR TB cases are included in Table 2.

**Table 2.** Onset of symptoms, and month and year of diagnosis and treatment initiation of extensively drug-resistant tuberculosis cases that are part of an outbreak linked to a university in Romania

XDR TB case number and country of origin	Month and year of symptom onset	Month, year, and place of TB diagnosis	Month, year and place of XDR TB diagnosis	Month, year and place of XDR TB treatment initiation	Type of disease (pulmonary/extra-pulmonary)
1 Israel	February 2015	July 2015 in Israel	September 2015 in Israel	October 2015 in Israel	Extra-pulmonary
2 UK	NA (asymptomatic; detected through contact tracing)	October 2015 in Romania: chest X-ray with lesions; December 2015 in Romania: GeneXpert: *Mycobacterium tuberculosis* and rifampicin resistance identified	March 2016 in Romania; March 2016 in the UK	March 2016 in UK	Pulmonary
3 UK	NA (asymptomatic; detected through contact tracing)	October 2015 in Romania: chest X-ray without lesions	October 2016 in the UK	October 2016 in the UK	Pulmonary
4 UK	March 2016	March 2016 in the UK	April 2016 in the UK	April 2016 in the UK	Pulmonary
5 Israel	NA (asymptomatic; detected through contact tracing)	October 2016 in Romania: chest X-ray with lesions; smear and culture negative	November 2016 in Israel	December 2016 in Israel	Pulmonary
6 Finland	June 2016	August 2016	September 2016 in Finland	August 2016 in Finland	Pulmonary
7 UK	NA (asymptomatic; detected through contact tracing)	September 2016	September 2016 in the UK	September 2016 in the UK	Pulmonary

NA, not applicable; TB, tuberculosis; XDR TB, extensively drug-resistant TB.

## Discussion

The investigation of this cross-border outbreak of XDR TB linked to a university in Romania, as the place of a possible transmission, identified seven confirmed cases. Four cases were detected in Israeli and British students of the university, and three were family members and other close contacts. WGS investigations showed that the isolates from six cases had identical WGS patterns, with zero SNPs difference, while the isolate from the index case showed just one SNP difference. Despite all the efforts at the national and international levels, no source case or source geographic area could be identified. The close collaboration between countries coordinated by ECDC enabled robust contact tracing and the prevention of further transmission across the EU.

XDR TB requires long and expensive treatment [8] and often has a negative treatment outcome [3]. Thus an outbreak of XDR TB warrants a rigorous investigation, both to identify additional cases and to identify the source. In this report, we provide information on the contact and the source investigation. The contact investigation used different approaches depending on the national guidelines and protocols and often combined diagnostic tests: TST, IGRA, chest X-ray, smear microscopy, Xpert MTB/RIF, and culture. For the source investigation 24 loci, MIRU-VNTR and WGS were used. MIRU-VNTR has been the standard method for studying transmission [9, 10]; more recently WGS has been applied for TB outbreak investigations [11]. Countries involved in this outbreak that routinely use WGS for TB investigations had the results rapidly available. In Israel, WGS is not routinely used and in Romania, it is not available; therefore, it took several months before the WGS link between the Israeli cases and the other cases could be confirmed. WGS is potentially an important new technology in the fight against infectious diseases. It provides the opportunity to identify TB transmission with high accuracy, next to information on drug resistance. In this specific outbreak investigation it confirmed links between different patients. Therefore, the establishment of WGS in all EU Member States may add to the quality and timeliness of international outbreak investigations, though the public health benefits still need to be more firmly established [12].

Contact investigation identified contacts with active and latent TB infection. All contacts with active TB received adequate (XDR) TB treatment. Options for prevention of TB among contacts of XDR TB patients are limited and require an individual risk assessment, taking into consideration the risk of progression to TB disease, drug susceptibility pattern of the source case and the risk of adverse drug events [13–16, 17]. Due to the resistance profile of the strain in this outbreak, there was no option for preventive treatment for identified contacts. Thus, they were put on clinical observation and provided with relevant information.

Country capacities for MDR TB detection have improved by about 40% since the inception of the Consolidated Action Plan to Prevent and Combat Multidrug – and Extensively Drug-Resistant Tuberculosis, in the WHO European Region, 2011–2015 [18]. In the EU 91% of all laboratory-confirmed TB cases were tested for at least isoniazid and rifampicin resistance in 2015 [3]. The improved capacity to diagnose drug resistance is important for identifying those who would not benefit from standard TB treatment. TB cases identified with drug resistance need to be put on an adequate treatment regimen at soonest so that the possibility of transmitting their drug-resistant strain is reduced as quickly as possible. The collaboration between the countries involved in this outbreak included the exchange of DST information which allowed for appropriate treatment to commence rapidly.

In 2015, 31,552 MDR TB cases were identified in the WHO European Region, of which 1242 in the EU. In the same year, EU countries reported 202 XDR TB cases with 77% of all MDR TB cases tested for resistance to second-line TB drugs. Even though a substantial number of XDR TB cases are diagnosed annually in the EU, only one report on an international investigation of an MDR TB cluster in the EU has been published [19]. Our report is, therefore, a first account on how countries can collaborate by exchanging information about XDR TB cases and about contacts and by attempting to find the source of the outbreak and inform about transmission.

The investigation was also important for European liaison to ensure that no infected medical students were working in healthcare in other EU countries. There are no general guidelines on the screening of returning medical students. However, in Israel, all healthcare workers are subjected to a screening which includes TST. The UK took the precaution of screening all students who had returned to the UK. No additional cases of TB were found. In Finland, all healthcare workers, who have risk factors for TB, are interviewed and screened by chest X-ray, before they can start working.

In the countrywide drug resistance survey in Romania in 2014–2015, cluster sampling method (20% sampling fraction), the proportion of MDR TB cases was 2.5% (38 out of 1494) among new cases and 10.8% (52 out of 481) among previously treated patients. Near 16% of the new MDR TB cases were diagnosed with XDR TB and 13.5% of the previously treated MDR TB cases had XDR TB. This shows that XDR TB is not a rare event in Romania (D. Homorodean, report not published). It is therefore unfortunate that we have not been able to identify the source of this outbreak since that may have provided valuable information as to where additional prevention and infection control activities would have been beneficial. However, since none of the cases in this outbreak was of Romanian origin it is also very possible that the strain was imported before it started circulating among the students at the university. It has already been demonstrated that migrants arriving from high TB-incidence countries may pose a significant challenge to TB control programmes in the host country [20].

We report on a cross-border outbreak investigation involving several countries. Each country followed specific country procedures for the investigation according to national regulations. This limits the comparability of the findings between countries. However, it illustrates the different approaches to cluster and contact investigations.

The international notifications helped to identify this outbreak and the international collaboration between epidemiologists, microbiologists and clinicians may have helped in the control of the outbreak by exchange of information and by discussing investigation and control approaches. It also enabled cases to be rapidly put on adequate treatment before individual drug resistance patterns were known. A well-defined mechanism for international coordination and collaboration would be of benefit for future cross-border TB outbreak investigations.

## Supplementary material

For supplementary material accompanying this paper visit http://doi.org/10.1017/S095026881800047X. click here to view supplementary material
