# Supplementary material for: Cross-border outbreak of extensively drug-resistant tuberculosis linked to a university in Romania
Source: Epidemiol Infect. 2018 May 17;146(7):824–31. doi: 10.1017/S095026881800047X (PMC9184956; doi:10.1017/S095026881800047X)
Supplement: Supplementary file 1 [file S095026881800047Xsup001.docx]

**CROSS BORDER OUTBREAK OF EXTENSIVELY DRUG RESISTANT TUBERCULOSIS LINKED TO A UNIVERSITY IN ROMANIA**

**Authors’ contributions**

**Odette Popovici** – Senior epidemiologist, MD in the Romanian NCSCCD, NFP for tuberculosis – Alternate, in relation with ECDC. She coordinated the data collection from the County Public Health Authority (CPHA), from the National Institute of Pulmonology (NIP) and from the National Reference Laboratory (NRL) for Tuberculosis Cluj-Napoca, she coordinated the development of this manuscript together with Philip Monk, acquired all needed data, prepared the draft of the manuscript and has approved the current version of the manuscript.

**Philip Monk**, FFPH Consultant in Health Protection, Public Health England, East Midlands Centre. He has coordinated the data collection from the UK, the development of this manuscript, having equal contribution with Odette Popovici, and has approved the current version of the manuscript.

**Daniel Chemtob** – Public Health Physician, MD, MPH, DEA. Director of the Department of Tuberculosis (TB) and AIDS and National TB Programme (NTP) manager, Jerusalem, Israel; and Senior Lecturer, Hebrew University-Hadassah Faculty of Medicine, School of Public Health and Community Medicine, Jerusalem, Israel. As the NTP manager of Israel, he initiated the data collection and coordinated the report of the two Israeli cases (including the first one who initiated all the current process), and of the epidemiological enquiries performed in Israel. He has participating in drafting the manuscript and has approved the current version of the manuscript.

**Domnica Chiotan** – Senior pulmonologist, MD, PhD in the Romanian National Institute of Pulmonology (NIP), NFP for tuberculosis – Member, in relation with ECDC. She delivered data from the national TB database to Odette Popovici, coordinated the creation of Protocol for tracing and follow-up of contacts with an XDR TB case, has participated in drafting the manuscript and has approved the current version of the manuscript.

**Paul Jeffrey Freidlin** – Ph.D., Coordinator for Molecular Biology, National Center for Mycobacteria, National Public Health Laboratories Tel Aviv. Responsible for extracting DNA, sending it for NGS sequencing, forwarding the NGS read sets to personnel investigating the cross border outbreak, genotyping the Israeli strains by 24 loci MIRU-VNTR and 43 spacer spoligotyping, constructing and maintaining the database of genotypes, and creating a new *Mycobacterium tuberculosis* cgMLST BioNumerics database which yielded results that agreed with the consortium findings of epidemiological closeness of the three Israeli isolates (2 from 1 student, 1 from the other student). He has participated in drafting the manuscript and has approved the current version of the manuscript.

**Ramona Groenheit** – Microbiologist, PhD, Director of WHO Supranational TB Reference Laboratory (SRL) in Stockholm at the Public Health Agency of Sweden, in relation with ECDC. She coordinated the whole genome sequencing and the analysis of all sequences in this outbreak, participated in drafting the manuscript and has approved the current version of the manuscript.

**Marjo Haanperä** – Senior researcher, PhD, National Institute for Health and Welfare, Helsinki, Finland. She is has preliminarily analysed the NGS data of the Finnish isolate and sent the data to this project. She has also checked the Finnish databases in search of the origin of the outbreak. She has participated in drafting the manuscript and has approved the current version of the manuscript.

**Daniela Homorodean** – Senior microbiologist, MD, PhD, director of the National Reference Laboratory (NRL) for Tuberculosis in Cluj-Napoca, Romania. She was responsible with selection, subculture and referral of strains to the SRL in Stockholm and sending laboratory data to Odette Popovici and Domnica Chiotan, has participated in drafting the manuscript, and has approved the current version of the manuscript.

**Mikael Mansjö** – Microbiologist, Public Health Agency of Sweden. Responsible for the whole genome sequencing of the Romanian isolates and the sequencing analysis of all whole genome sequences produced in the UK, Finland and Israel. He has participated in drafting the manuscript and has approved the current version of the manuscript.

**Esther Robinson-** Lead Public Health Microbiologist for East Midlands and Consultant Microbiologist, National Mycobacterial Reference Service, National Infection Service, Public Health England. She was responsible with the National Mycobacterial Reference Service in the Midlands Public Health Laboratory for whole-genome sequencing of the UK strains. She has participated in drafting the manuscript and has approved the current version of the manuscript.

**Efrat Rorman** – Ph.D., Director of National Public Health Laboratories, Tel Aviv, Israel which includes the National Mycobacterium Reference Center. As Head of the National Public Health Laboratories Tel Aviv, she coordinated all resources used for isolation, growth, identification, drug sensitivity testing, DNA sequencing and forwarding of critical data to personnel collecting, analyzing and summarizing data on the cross border outbreak. She has participated in drafting the manuscript and has approved the current version of the manuscript.

**Grace Smith** - Consultant Clinical Microbiologist, Director National Mycobacterial Reference Service, National Infection Service, Public Health England. She was responsible with the National Mycobacterial Reference Service in the Midlands Public Health Laboratory for Whole Genome Sequencing of the UK strains and has participated in drafting the manuscript and has approved the current version of the manuscript.

**Hanna Soini** – Senior Expert, PhD, National Institute for Health and Welfare, Helsinki, Finland. She coordinated the outbreak investigation in Finland and has approved the current version of the manuscript.

**Marieke J. van der Werf** – Head of Disease Programme Tuberculosis, MD, PhD, MSc, MPH, European Centre for Disease Prevention and Control (ECDC), Stockholm, Sweden. She coordinated the cross border outbreak investigation for ECDC, proposed the outline of the manuscript, supervised the drafting of the manuscript and contributed substantially to the discussion section. She approved the current version of the manuscript.

**Acknowledgements**

We would like to acknowledge the Romanian epidemiologists and clinicians involved in this outbreak investigation, with special thanks to Dr. Mariana Fericean, senior epidemiologist in the county public health authority and Dr. Marcela Vigdorovits, senior pulmonologist in the local TB Dispensary; the Israeli epidemiologists and clinicians involved in this outbreak investigation, with special thanks to the Dr. Ahmed Attamna, head of the TB regional center in Hadera, to Dr. Bella Shulman from the Hadera sub-district health office, to Dr. Haytam Namarna from the TB center in Nazareth, and to Dr. Daniele Bendayan, head of the National TB ward; the staff of the National Center for Mycobacteria in Israel, especially the Director, Dr. Hasia Kaidar-Shwartz; the staff of the National Public Health Laboratories, Israel, with special thanks to Dr. Israel Nissan for sequencing and bioinformatics support; Dr. Erik Alm at the Public Health Agency of Sweden for his bioinformatics support and valuable discussions regarding the analysis of all WGS results and MSTs; Dr. Matthias Merker, German Center for Infection Research, Borstel Site, Borstel, Germany, for comparing the outbreak strain with the databases available in Borstel; the researchers in the Modernising Medical Microbiology consortium at the University of Oxford, UK with special thanks to Dr Timothy Walker, Clinical Lecturer in Infectious Diseases; and the experts of the European Centre for Disease Prevention and Control Sergio Brusin, Denis Coulombier, Vahur Hollo, Josep Jansa, Csaba Ködmön, Otilia Mardh, and Brigita Molnarova for their contribution to the outbreak investigation.
